# Supplementary material for: Causal Relationship between Aging and Anorexia Nervosa: A White-Matter-Microstructure-Mediated Mendelian Randomization Analysis
Source: Biomedicines. 2024 Aug 16;12(8):1874. doi: 10.3390/biomedicines12081874 (PMC11351342; doi:10.3390/biomedicines12081874)
Supplement: Supplementary file 1 [file biomedicines-12-01874-s001.zip › Supplementary Figure/Figure S9/Leaveoneout plot of MR analysis for ubm-b-1643 to AN.pdf]

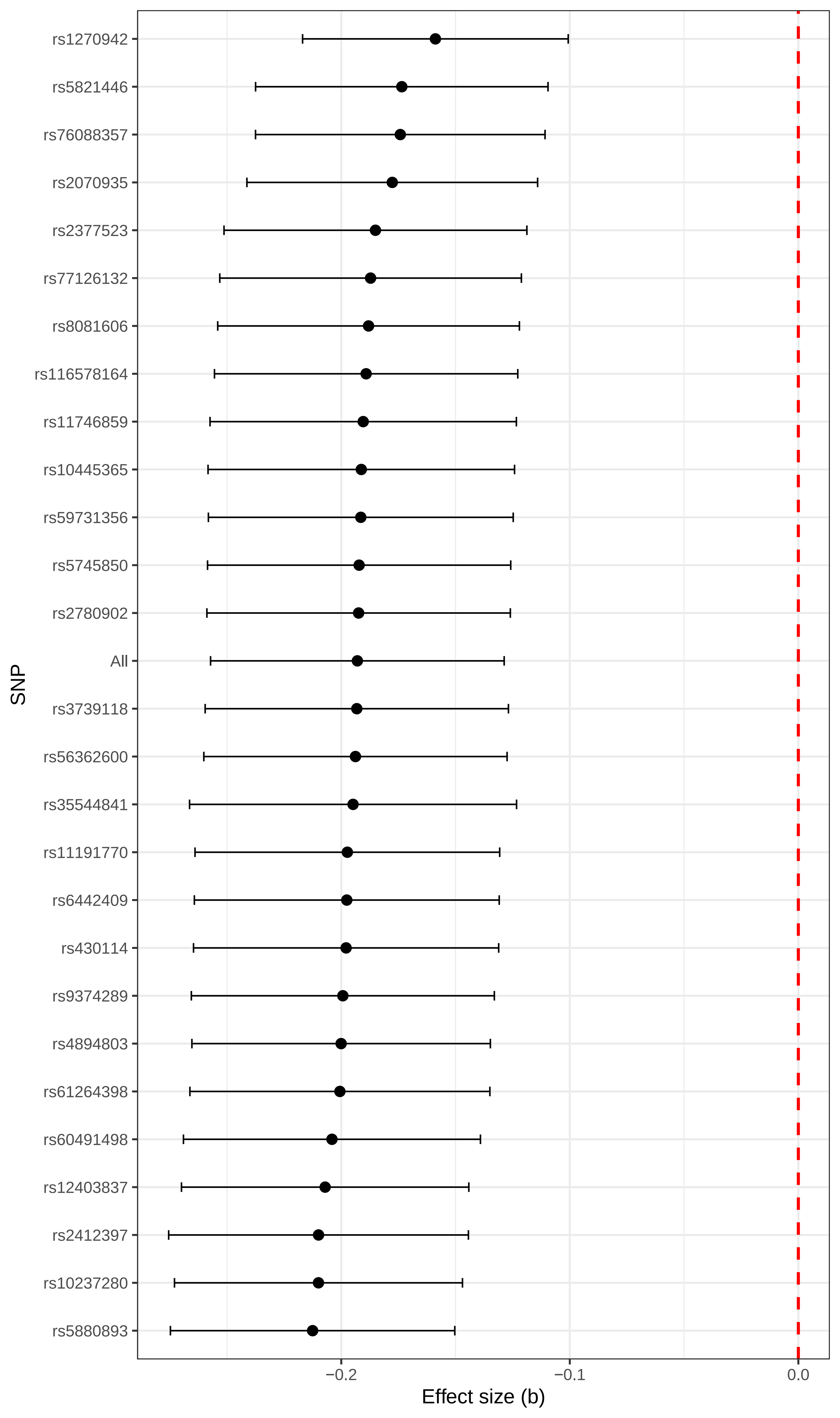

rs1270942
rs5821446
rs76088357
rs2070935
rs2377523
rs77126132
rs8081606
rs116578164
rs11746859
rs10445365
rs59731356
rs5745850
rs2780902
All
rs3739118
rs56362600
rs35544841
rs11191770
rs6442409
rs430114
rs9374289
rs4894803
rs61264398
rs60491498
rs12403837
rs2412397
rs10237280
rs5880893
SNP
−0.2
−0.1
0.0
Effect size (b)
